# The Expression of CD28 and Its Synergism on the Immune Response of Flounder (*Paralichthys olivaceus*) to Thymus-Dependent Antigen

**DOI:** 10.3389/fimmu.2021.765036

**Published:** 2021-11-11

**Authors:** Jing Xing, Wenjing Liu, Xiaoqian Tang, Xiuzhen Sheng, Heng Chi, Wenbin Zhan

**Affiliations:** ^1^ Laboratory of Pathology and Immunology of Aquatic Animals, Key Laboratory of Mariculture, Ministry of Education (KLMME), Ocean University of China, Qingdao, China; ^2^ Laboratory for Marine Fisheries Science and Food Production Processes, Qingdao National Laboratory for Marine Science and Technology, Qingdao, China

**Keywords:** CD28, keyhole limpet hemocyanin and phytohemagglutinin, lipopolysaccharide, T cell activation, *Paralichthys olivaceus*

## Abstract

CD28 is well known as a critical T-cell costimulatory receptor involved in T cell activation by binding to its ligands. In this study, CD28 was cloned, and its expression profiles were characterized in flounder (*Paralichthys olivaceus*); variations of CD28+ cells after being stimulated with different types of antigens and the function of the CD28 costimulatory pathway on T-cell activation were investigated *in vitro*. *fCD28* consists of four exons and three introns, and the full-length cDNA of *fCD28* was 675-bp encoded 224 amino acids. The conserved motif (^121^TFPPPF^126^) binding to the CD80/86 ligand exists in the Ig-superfamily homology domain. The high expression of *fCD28* is in gills, PBLs, head kidney, and spleen. CD28+ cells were co-localized with CD4+ T lymphocytes but not on IgM+ B lymphocyte cells. Moreover, the expression of CD28 was significantly varied in flounder after being stimulated by keyhole limpet hemocyanin (KLH) at both the transcriptional and cellular levels, while no significant differences were observed between lipopolysaccharide (LPS) stimulation and the control group. Notably, treatment of PBLs cultured *in vitro* with CD28 molecule-specific antibody (anti-CD28 Abs) and PHA produced more cell colonies and stimulated the proliferation of cultured leukocytes compared to PHA stimulation alone and the control group, and a higher level of IL-2 was detected in the culture medium. Meanwhile, anti-CD28 Abs increased the percent of CD28+ cells (10.41 ± 1.35%), CD4+ T lymphocytes (18.32 ± 2.15%), and CD28+/CD4+ double-positive cells (6.24 ± 1.52%). This effect also resulted in significant variations in the genes of cell membrane-bound molecules, cytokines, and related signaling pathways in cultured leukocytes, with significant changes in the genes of *interleukin-2 (IL-2)* and *nuclear factor of activated T cells (NFAT)* in the early stages of culture, and the expression of other molecules increased over time. These results proved the localization of the CD28 molecule on T lymphocytes in flounder, and anti-CD28 may act as the B7 ligand involved in T cell activation after antigen stimulation. These data provide a basis for a more in-depth study of the mechanism of the CD28 costimulatory pathway in T cell activation.

## Introduction

T lymphocytes as an important type of leukocyte are an integral and essential part of the host cellular immune response (1). Cells could directly kill cells that are infected by viruses and other intracellular microbes or release signal molecules to recruit macrophages to destroy phagocytosed invading microbes (2). In other cases, activating T cells could also release cytokines and help B lymphocytes to produce antibodies (1, 3). Full activation of naïve T cells is a complex process; only the combination of the T cell receptor (TCR) with antigenic peptide complexed with major histocompatibility complex (MHC) is insufficient. Indeed, the engagement of the second signal has been confirmed to decrease the threshold of T cell activation and avoid the phenomenon of T cell anergy (4–6).

CD28, a best-characterized co-stimulation molecule expressed on T cells, is a type I transmembrane glycoprotein of the Ig superfamily and constitutively expressed on the surface of 80% CD4^+^ T cells and 50% CD8^+^ T cells in the human model and 100% on both CD4^+^ and CD8^+^ T cells in the mouse model (7, 8). Structurally, the CD28 monomer usually consists of a signal peptide, an extracellular V-set immunoglobulin superfamily (IgSF) domain, a transmembrane region, and a cytoplasmic tail and functions as a glycosylated, disulfide-linked homodimer. CD28 interacts with the B7 molecule expressed on antigen-presenting cells (APCs, including macrophages, dendritic cells, B lymphocytes) through the conversed motif “MYPPPY” within the IgV-like domain (9, 10). The B7 family contains two members-B7-1 (CD80) and B7-2 (CD86); they both bind to CD28 molecules through the MYPPPY motif, but their binding abilities are different, with CD80 binding more strongly to CD28 than CD86 (11). However, cytotoxic T-lymphocyte-associated protein 4 (CTLA-4), a molecule highly similar to CD28, performs the opposite immunological function of CD28. CTLA-4 binds the B7 molecule with a higher affinity than CD28 does, thereby maintaining homeostasis by competitively binding CD28 ligands and inhibiting T-cell activation (12, 13). The role of CD28 molecules in T cell activation is complex. When the T cells receive the first signal (peptide-MHC complex) delivered by APCs, the intracellular tyrosine within the conversed motif “YMNM” of CD28 bound to the B7 molecule is phosphorylated (8). The p85 regulatory subunit of PI3K is recruited, and binding to the “YMNM” motif in turn activates PI3K. Activated PI3K converts phosphorylation of phosphatidylinositol biphosphate (PIP2) into phosphatidylinositol 3-phosphate (PIP3) which promotes activation of protein kinase B (PKB/Akt), and then PKB/Akt phosphorylates its downstream functional molecule including mTOR, IκB, and GSK3β (14–16). Finally, CD28 cooperates with TCR leading to the transcriptional activation of NFAT, AP-1, and NF-κB transcription factors. The activation of NF-κB and NFAT induces upregulated transcription of both Bcl-xL, which favors T-cell survival, and IL-2, which is an important T-cell cytokine required for proliferation (17). Additionally, the CD28 signal also activates the JnK cascade, and activated JnK phosphorylates transcription factors Jun, thereby activating the AP-1 complex to regulate cell proliferation (18, 19). Based on the important role of CD28 as costimulatory molecules, the combined use of CD28 and CD3 antibodies is the best method for activating T cells *in vitro* in humans (20–23), although many non-specific mitogens like PHA (phytohemagglutinin) (24, 25) or Con A (Concanavalin A) also provide activation signals to T cells in different ways (26).

Given the fundamental position of fish in the vertebrate phylogeny, the study of their immune system has gained more attraction. Current studies have demonstrated that the basic components of the mammalian immune system (B and T lymphocytes, MHC, CDs, cytokines, etc.) are also present in fish (27, 28). Recently, more CD28 homologs had been identified in teleost including Half-smooth tongue sole (*C. semilaevis*) (29), Nile tilapia (*O. niloticus*) (30), European sea bass (*D. labrax*) (31), rainbow trout (*O. mykiss*) (32), river pufferfish (*T. obscurus*) (33), and rock bream (*O. fasciatus*) (34). The authors have confirmed that CD28 molecules were constitutively expressed in various tissues of fish and they show different expression profiles in response to stimulation by bacterial or viral stimuli. Moreover, Huang *et al.* describe in their study that CD28 molecules can bind to CD80/86 molecules at the protein level in tilapia (30). In half-smooth tongue sole, the CD28 polyclonal antibody was able to proliferate head kidney lymphocytes and cause upregulation of IL-2 expression (29). Therefore, given these observations, it has been suggested that CD28 molecules may play a similar function as a CD28 homolog in mammals.

However, these studies have not delineated the distribution characteristics of the CD28 molecule in different types of lymphocytes in fish and their response characteristics to KLH, PHA, and LPS which are usually distinguished by the need for T-cell involvement in the induction of an immune response (35, 36). In the present study, we aim to elucidate the structural features, distribution of lymphocytes, and importance in T/B cell immune response. Here we cloned the CD28 homolog from flounder (*Paralichthys olivaceus*) and characterized the expression in different tissues. Further, the distribution characteristics of CD28 molecules in different leukocyte subpopulations were investigated by the prepared anti-CD28-specific antibodies. In addition, we also confirmed that the co-action of PHA with specific antibodies to CD28 molecules is associated with T cell activation. Those results provide more basic features and functions of the CD28 and provide a promising approach to T activation in teleost.

## Materials and Methods

### Animals, Cells, and Antibodies

Healthy flounder (*P. olivaceus*) of 50 ± 5.8 g or 1 ± 0.1 kg were purchased from a farm in Rizhao, Shandong Province, PR China, and maintained in the aerated recirculating seawater system at 20 ± 2°C for 2 weeks. The flounder with no clinical symptoms determined by general appearance and level of activity were used in the following experiments.

New Zealand white rabbits (∼1 kg) and Balb/C (~30 g) mice were purchased from Qingdao Animal Experimental Center (Shandong, China) and then used for antibody production.

HINAE cells, provided by Dr. Ikuo Hirono of Tokyo University of Marine Science and Technology (37), were cultured in Leibovitz’s L-15 medium containing 10% FBS, 100 IU/ml penicillin, and 100 μg/ml streptomycin.

Mouse polyclonal antibodies against flounder IL-2 (diluted at 1:500) and monoclonal antibodies against flounder IgM (diluted at 1:1,000), CD4-1 (diluted at 1:1,000), and CD4-2 (diluted at 1:1,000) were previously produced in our laboratory (38–40). The secondary antibodies included DyLight 488 (Abbkine; diluted at 1:1,000) rabbit or mouse IgG, DyLight 649 (Abbkine; diluted at 1:1,000) rabbit or mouse IgG, alkaline phosphatase (AP)-conjugated goat anti-rabbit IgG (H + L) (Abbkine; diluted at 1:5,000), and HRP-conjugated goat anti-mouse IgG (Abbkine; diluted at 1:5,000).

### Gene Cloning of CD28 in Flounder

Total RNA from tissues (spleen and head kidney) was extracted from flounder using TRIzol Reagent (Invitrogen, USA) and the first-strand synthesis using PrimeScript™ First-Strand cDNA Synthesis Kit (Takara, China). Total Genomic DNA from head kidney was extracted using a TIANamp Marine Animals DNA Kit (Tiangen, China) according to the manufacturer’s instructions. The CD28 sequences of *C. semilaevis*, *O. niloticus*, or *D. labrax* were used to search the flounder transcript database published on the National Center for Biotechnology Information (NCBI; http://www.ncbi.nlm.nih.gov) through BLASTn or BLASTp. Based on the partial sequence searched, specific primers were designed by Primer Premier 5.0 (listed in **Table 1 of the Supplemental Material**) to extend the 3′ and 5′ untranslated region (UTR) using cDNA from the spleen by the rapid amplification of cDNA ends (RACE) method. All PCRs were performed in a 50-μl reaction containing Ex Taq 0.25 μl, 10× Ex Taq buffer 5 μl, dNTP mixture 4 μl, forward primer 1.5 μl (10 μM), reverse primer 1.5 μl (10 μM), cDNA 2 μl, and DEPC H_2_O 35.75 μl. The thermocycling program was 98°C for 1 min, 35 cycles of 98°C for 10 s, 60°C for 30 s, and 72°C for 1 min, followed by a final extension period of 72°C for 5 min. The PCR products were electrophoresed on 1% agarose gels, and the expected segment was extracted using EasyPure^®^ Quick Gel Extraction Kit (TransGen, China) and cloned into the pClone007 Simple Vector (Tsingke, China). Following transformation into competent *E. coli* DH5α cells (TransGen, China), positive clones were screened by ampicillin selection and colony PCR and then sequenced by Tsingke Biological Technology (Qingdao, China).

### Sequence Analysis

The full-length CD28 cDNA was assembled by DNAman and mapping the intron/exon composition of the CD28 molecule by comparing the genomic CD28 sequence with the CD28 cDNA sequence obtained by cloning. The potential open reading frame (ORF) was analyzed with the Finder program (https://www.ncbi.nlm.nih.gov/orffinder/). The protein analysis was conducted with the ExPASy tools (http://expasy.org/tools/). The signal peptide and the TM domain of the deduced protein sequences were predicted with the programs SignalP (http://www.cbs.dtu.dk/services/SignalP/) and TMHMM (http://www.cbs.dtu.dk/services/TMHMM/), respectively. Multiple-sequence alignment was performed with the ClustalX program and the DNAman software. Phylogenic trees were constructed using the neighbor-joining method with MEGA software (Version 6.0) and were bootstrapped 1,000 times. Phosphorylation sites were predicted with the NetPhos 2.0 Server (http://www.cbs.dtu.dk/services/NetPhos-2.0/). Secondary and 3D structures were analyzed using SMART, SWISS-MODEL, and I-TASSER (https://zhanglab.ccmb.med.umich.edu/I-TASSER/).

### Expression Profiles of the CD28 Gene

A total of 18 flounder (50 ± 5.8 g) were used for detecting the expression of fCD28 in various tissues. Seven tissues including intestine, liver, PBLs, muscle, head kidney, spleen, and gills were sampled from nine healthy fish killed by overdose benzocaine (Sigma-Aldrich). To detect the variety of *CD28* after being injected with KLH (Sigma, USA), a total of 180 flounder (50 ± 5.8 g) were intraperitoneally injected with 200 μg/fish KLH and the control group was injected with an equal amount of PBS. PBLs, head kidney, and spleen were randomly collected from nine individuals in each group at various periods. All the tissues had been flushed by PBS to remove blood. The expression levels were examined by real-time qPCR.

### Preparation of Recombinant Protein and Production of Antibodies

Primers used to amplify the CD28 sequence without the signal peptide and amplify the full length of CD28 are listed in **Table 1** (in the **Supplemental Material**). The PCR product was purified and ligated to the *Hind*III and *BamH*I restriction enzymes of pET-28a vector (TaKaRa, Japan) or pTagRFP-N vector (Evrogen, Russia). Then, the recombinant prokaryotic plasmids were transformed into *Escherichia coli* BL21 Star (DE3) and induced by adding 1 mM isopropyl thiogalactoside (IPTG) for 12 h at 37°C. The recombinant proteins with a 6-Histidine tag at the N-terminus were affinity-purified using His Trap™ HP Ni-Agarose (GE Healthcare China, Beijing, China). The recombinant eukaryotic plasmid was extracted using the EndoFree plasmid Kit (Tiangen, China) following the manufacturer’s protocol. Then, the concentration of the plasmid was adjusted to 500 ng/μl with sterile PBS for transfection.

The purified recombinant protein was resuspended in sterile PBS. New Zealand white rabbits and Balb/c mice were immunized with the recombinant CD28 recombinant proteins (600 μg for rabbits and 50 μg for mice, respectively) in Complete Freund’s adjuvant (Sigma, USA) on day 1 and in Incomplete Freund’s adjuvant (Sigma, USA) on days 14, 28, and 42. Five days after the final immunization was administered, antiserum was collected from animals. After being purified, the Abs titers were determined by ELISA, and the specificity was characterized by Western blot.

### Variation of CD28^+^ Cells in Flounder After Stimulation of KLH and LPS

A total of 240 healthy flounder (50 ± 5.8 g) were used for detecting the variation of CD28^+^ cells in flounder after stimulation of KLH and LPS. Flounder were randomly divided into three groups with 120 fish in each one. One of the immunized groups was intraperitoneally injected with 200 μg/fish KLH. The other immunized group was also immunized with 200 μg/fish lipopolysaccharide (LPS, Sigma, USA), a thymus-independent antigen. As the control group, fish were injected with the same volume of PBS. The leukocytes in peripheral blood were isolated by discontinuous Percoll (Pharmacia) gradient (1.020/1.070) on the 1st, 3rd, 5th, 7th, 9th, 11th, and 14th days post-injection (39). Changes in CD28+ cells were analyzed by flow cytometry.

### The Culture of PBLs Treated Using PHA and Anti-CD28 *In Vitro*

Leukocytes from peripheral blood were isolated in sterile conditions, as mentioned above. In order to avoid the influence of interactions between cells from different fish on the experimental results, PBLs from one fish were used for each experiment, and each experiment was repeated three times. Cells were adjusted to 1 × 10^6^ cells/ml after being washed three times with PBS to remove serum. Resuspended cells were labeled with carboxyfluorescein diacetate succinimidyl ester (CFSE, Invitrogen) to a final 2-μM concentration and immediately incubated for 10 min in the dark. Straining was terminated by washing the cells with an ice-cold L-15 medium containing 10% FBS and incubated in ice for 5 min. Then, cells were washed three times in L-15 medium with 10% FBS and adjusted to 5 × 10^6^ cells/ml and placed into a 12-well culture plate (2 ml/well) cultured in L-15 medium with 10% FBS. Stimulated cells were treated with 5 μg/ml PHA (Sigma) or 5 μg/ml PHA + 0.5 μg/ml anti-CD28 Abs (40). Unstimulated cells were treated with an equal amount of sterile PBS medium as negative controls. To verify whether anti-CD28 Abs could similarly induce lymphocyte proliferation as in mammals, the cells were collected at 72 h and the division was analyzed by flow cytometry. The cultured cells were harvested at 0, 8, 24, and 36 h to detect the expression levels of immune-related genes which were examined by RT-qPCR. Supernatants of cultured peripheral blood leukocytes were also collected at 72 h. After concentration and quantification to the same concentration (2~3 mg/ml), Western blotting was conducted to detect the IL-2 level; mouse anti-IL-2 antibodies were used as primary antibodies. The ImageJ software (V1.8.0) was used to process and analyze images. Each culture was performed in duplicate.

### Western Blotting

When the density of cultured HINAE cells reached about 80%, the cells were transfected with 500 ng recombinant eukaryotic plasmid pTagRFP-N-CD28 or pTagRFP-N using Lipofectamine^®^ 3000 (Thermo Fisher, MA, USA) following the manufacturer’s instructions. When cells were cultured until a clear fluorescent signal could be detected, the cells were collected. Purified recombinant proteins, whole-cell protein extracts from PBLs, or transfected HINAE cells were applied to Western blotting. The primary antibody was detected with alkaline phosphatase (AP)-conjugated goat anti-rabbit IgG (H + L) or HRP-conjugated goat anti-mouse IgG (H + L). Non-immunized serum instead of primary antibodies was used as a control.

### Immunofluorescence Staining and Flow Cytometry

Leukocytes from head kidney, spleen, and peripheral blood were isolated. After being adjusted to 5 × 10^6^ cells/ml in PBS, leukocytes including cultured leukocytes were incubated with anti-CD28 Abs (rabbit or mouse) or for 1.5 h at 37°C. Unimmunized rabbit or mouse serum was used as the negative control. Then the cells were incubated with secondary Abs for 1 h at 37°C in the dark. Flow cytometry was performed using FACSCalibur (BD Biosciences) flow cytometers with acquisition enabled by CellQuest Pro software (BD Biosciences). Data were analyzed using FlowJo 10.7 (TreeStar). For immunofluorescence observation, 20 μl cell suspension (1 × 10^6^ cells/ml) was fixed onto APES-coated slides. After sequential incubation of primary and secondary antibodies, leukocytes were counterstained with 4,6-diamidino-2-phenylindole (DAPI) to confirm nuclear staining and then observed under a fluorescent microscope (Olympus DP70, Japan).

### Real-Time qPCR

RT-qPCR was performed using the LightCycler^®^ 480 II Real-Time System (Roche, Switzerland). Each reaction consisted of 10 μl 2×ChamQ Universal SYBR qPCR Master Mix (Vazyme, China), 0.4 μl forward primer and 0.4 μl reverse primer (10 μM), 2 μl cDNA, and 7.2 μl DEPC H_2_O. The qPCR primers and housekeeping gene are listed in **Table 2** (in the **Supplemental Material**), and the amplification fragment size of the primers was between 150 and 250 bp and the amplification efficiency of the primers was confirmed to be within 90%–110%. The β-actin gene was used as an internal control. The 2^−ΔΔCT^ method was used to analyze the expression level of genes.

### Statistics

Each experiment was repeated at least three times. Statistical analyses were performed using Statistical Product and Service Solution (SPSS) 20.0 software (IBM, Armonk, NY, USA) with one-way analysis of variance (ANOVA) followed by t-test or Duncan’s multiple-range test. All data are expressed as the mean ± standard deviation (SD). Statistical parameters and details of experiments are provided in the figure legends, and exact *p* values are shown in the figures. GraphPad Prism was used for plotting graphs.

## Results

### Sequences and Its Homology of fCD28

**Figure 1:**
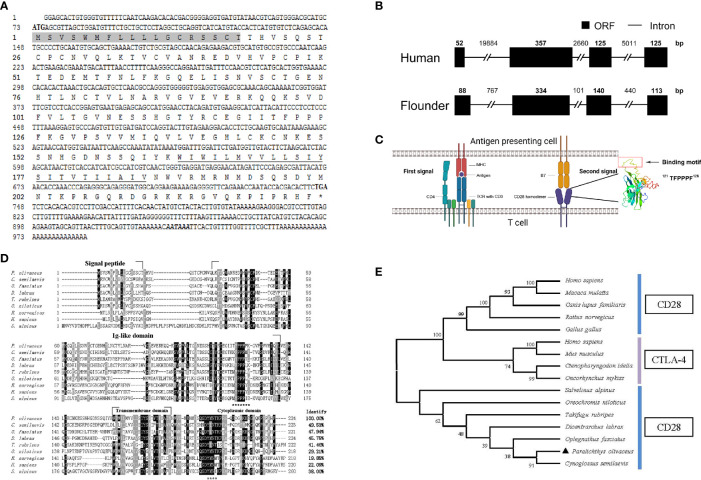
Cloning the *CD28* gene and bioinformatics analysis of deduced amino acid sequence in flounder. **(A)** The cDNA sequence and deduced amino acid of *CD28*. The signal peptide is shadowed, and the transmembrane domain is underlined. The asterisk indicates the stop codon. Putative polyadenylation signal (*AATAAA*) is in bold italic. **(B)** Comparison of the intron/ORF structures of the *CD28* gene of human and flounder. ORF and introns are shown with black boxes and lines, respectively. The sizes of the intron/ORF are indicated by the number above. **(C)** Schematic diagram of the two-signaling pattern of T cell activation and tertiary structure of the extracellular domain of CD28 molecule. **(D)** Multiple alignments of CD28 protein homologs. The residues that are ≥75% identical among the aligned sequences are in black. The conversed motifs are indicated with asterisks below the alignment. The dashes in the amino acid sequences indicate gaps introduced to maximize alignment. **(E)** Phylogenetic trees of CD28 and CTLA-4 molecules homologs. The unrooted phylogenetic trees are constructed by the neighbor-joining method based on the amino acid alignment (Clustal W) of full-length protein sequences. Numbers in each branch indicate the percentage bootstrap values on 1,000 replicates. The accession numbers used in **Figure S3** are listed in **Supplementary Materials**.

The cloned full-length cDNA of *fCD28* in flounder consists of 986 bp with a 72-bp 5′ untranslated region (UTR), a 675-bp ORF, and a 387-bp 3′-UTR (GenBank accession no. MT019836.1) (**Figure 1A**). By comparing cDNA sequences with gene sequences, the results showed that the *fCD28* gene comprised four exons and three introns. It might be worth noting that the genomic structure is highly conserved with the human CD28 gene (**Figure 1B**). The ORF sequence encoded a protein of 224 amino acids with a putative molecular mass of 25.3 kDa, and the predicted isoelectric point was 8.89. The amino acid sequence alignment shows that CD28 shares overall 19.86%–49.53% amino acid identity with other vertebrates’ counterparts (**Figure 1D**). To further analyze the evolutionary relationship of CD28 molecules between fish and mammalian, the N-J tree was constructed with bootstrap analysis (1,000 replicates); the results showed that CD28 of flounder clustered into one group in teleost with high bootstrap support and demonstrated exclusivity with CTLA-4 which exerted a co-inhibitory effect through binding to B7 molecules (**Figure 1E**).

### Protein Structure of fCD28

The structural features were analyzed using SMART (http://smart.embl-heidelberg.de/); as a typical type I transmembrane protein, the structure of the CD28 molecule in flounder is composed of a signal peptide, a superfamily Ig domain in the extracellular domain, a transmembrane domain, and a cytoplasmic region. As in other species, the CD28 molecule in flounder also contains a conserved binding motif ^121^TFPPPF^126^ responsible for binding to B7 molecules in the extracellular Ig-like domain. In parallel, another motif ^199^YMNT^202^ appears in the cytoplasmic tail responsible for signal transduction through phosphorylation. Meanwhile, tyrosine phosphorylation sites were found to be present in the “YMNT” motif. Through homologous modeling using the human CTLA-4 extracellular domain as templates (PDB Hit: 1AH1, C-score = -3.66, TM-score = 0.31 ± 0.10), a three-dimensional model was constructed using the extracellular region of the CD28 molecule in flounder, and the motif responsible for binding to the B7 molecule is present in the 3D structure in a linear fashion, as shown in **Figure 1C**. Altogether, the CD28 molecule was cloned and it shared similar sequences and structural features to homologs in other species.

### Gene Expression Profiles of fCD28

**Figure 2:**
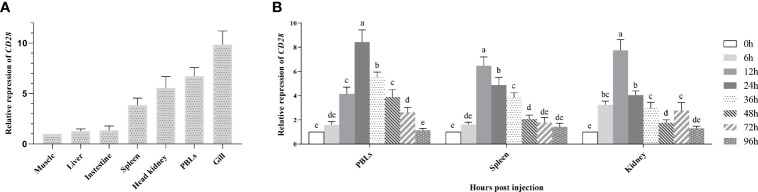
Gene expression analysis. **(A)** Expression of *CD28* in flounder tissues. Relative expression levels of *CD28* in different tissues were normalized first concerning the expression level of β-actin and then compared with the average expression level in the muscle where the expression level was lowest and defined as 1, n=9. **(B)** Induced expression analysis of *CD28* after KLH stimulation in PBLs, spleen, and head kidney. The relative expression values were averaged from the data in three parallel reactions, and the results were obtained from at least three independent experiments, n = 9. Different letters above the bar represent the statistical significance (*p* < 0.05). Error bars represent standard errors of SD.

To portray the expression profile of f*CD28* at the transcriptional level, real-time RT-PCR (RT-qPCR) was performed to detect *CD28* in different tissues. As shown in **Figure 2A**, *CD28* was constitutively expressed in each of the tissues examined and was strongly expressed in immune-relevant tissues including gill, PBLs, head kidney, and spleen. Among them, the highest expression was in gills. In contrast, the expression was lower in muscle, liver, and intestine, and there was almost no difference in the expression among the three tissues.

After KLH stimulation, the transcriptional change of *fCD28* in PBLs, spleen, and head kidney was determined by RT-qPCR using *β-actin* as a housekeeping gene. Compared to the control time point (0 h), the expression of *CD28* gradually increased and reached the peak value at 24* h* and presented a significant difference (ANOVA, *p* < 0.05) during 12–72 h in PBLs; in the spleen, *fCD28* expression levels reached the maximum at 12 h which was earlier than in PBLs and presented significant difference (ANOVA, *p* < 0.05) during 12–48 h. The mRNA expression in the head kidney also reached the highest expression level at 12 h post-stimulation, and the expression of *fCD28* decreased at 36 h and then showed differences (ANOVA, *p* < 0.05) from the control group at 72 h (**Figure 2B**).

### Antibody Specificity and Subcellular Localization of CD28 in Flounder

**Figure 3:**
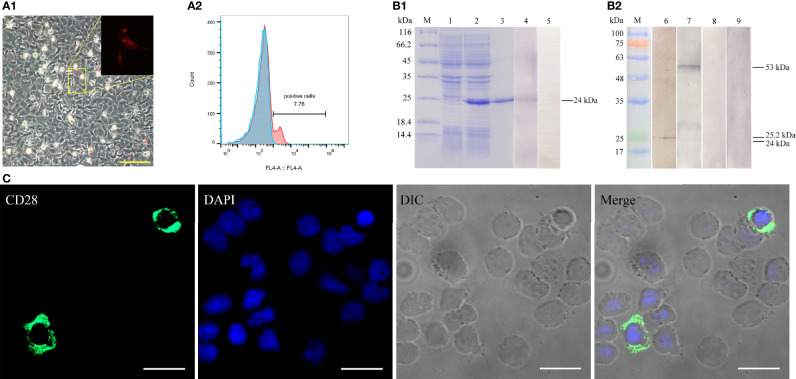
Verification of the specificity of the prepared antibodies. **(A)** Detection of CD28 expression in transfected HINAE cells. A1, The red signal means that HINAE transfected with pTagRFP-N-CD28 plasmid successfully expressed the recombinant protein. Scale bar = 50 μm. A2, Flow cytometric analysis of the percentage of cells expressing CD28 after transfection. **(B)** Western blotting analysis of anti-CD28 rabbit polyclonal antibody specificity. Lane M, molecular mass marker; lane 1, transformed *E. coli* without IPTG induction; lane 2, transformed *E. coli* induced with IPTG; lane 3-5, purified recombinant CD28 protein (24 kDa); lanes 6 and 8, whole-cell protein extracts from PBLs; lanes 7 and 9, whole-cell protein extracts from HINAE cells transfected with pTagRFP-N-CD28 vector. **(C)** Subcellular localization of CD28 on PBLs. CD28 molecule mainly located on the cell surface. Scale bar =10 μm.

The recombinant CD28 protein (~23.2 kDa) with His-tag (~0.84086 kDa) was purified and analyzed by SDS-PAGE. As shown in **Figure 3B1**, a highly purified recombinant CD28 protein with the predicted molecular mass was obtained. Rabbit/mouse anti-CD28 polyclonal antibodies were produced, and the rabbit anti-CD28 Abs were purified by using affinity purification (**Figure S1 in the supplemental material**). The titers of the prepared rabbit and mouse anti-CD28 polyclonal antibodies were analyzed by ELISA. The results showed that the polyclonal antibody had average titers higher than 1:100,000 (data not shown).

To verify that the natural CD28 protein could be recognized by the prepared polyclonal antibodies, the constructed pTagRFP-N-CD28 vector was transfected into the HINAE cell line. The recombinant CD28 protein with RFP-tag was successfully expressed in the HINAE cell line (**Figure 3A1**) with approximately 7.8 ± 1.5% of the transfection percentage analyzed by flow cytometry (FCM) (**Figure 3A2**). The Western blotting results showed that the prepared polyclonal antibody could recognize the purified CD28 protein at the molecular weight of 24 kDa with a clear reaction band (**Figure 3B1** **, lane 4**). Moreover, Abs could also react with protein’s molecular weight of 25.2 and 53 kDa with PBLs or transfected HINAE cell crude extracts, respectively (**Figure 3B2** **, lanes 2-3**), which is consistent with the molecular weight of the CD28 natural protein. No bands were observed in the negative control groups (**Figure 3B** **, lanes 5, 8, 9**). As the result of the structural analysis of CD28, the positive signal was detected mainly on the membrane surface of leukocytes after incubation with the prepared polyclonal antibody (**Figure 3C**) in immunofluorescence assays. The results suggest that the prepared antibodies showed specific recognition of the CD28 molecule, and CD28 is expressed in a membrane-bound form on the surface of lymphocytes as mammals.

### Percentage of CD28^+^/CD4-1^+^, CD28^+^/CD4-2^+^, and CD28^+^/IgM^+^ Cells in Tissues

**Figure 4:**
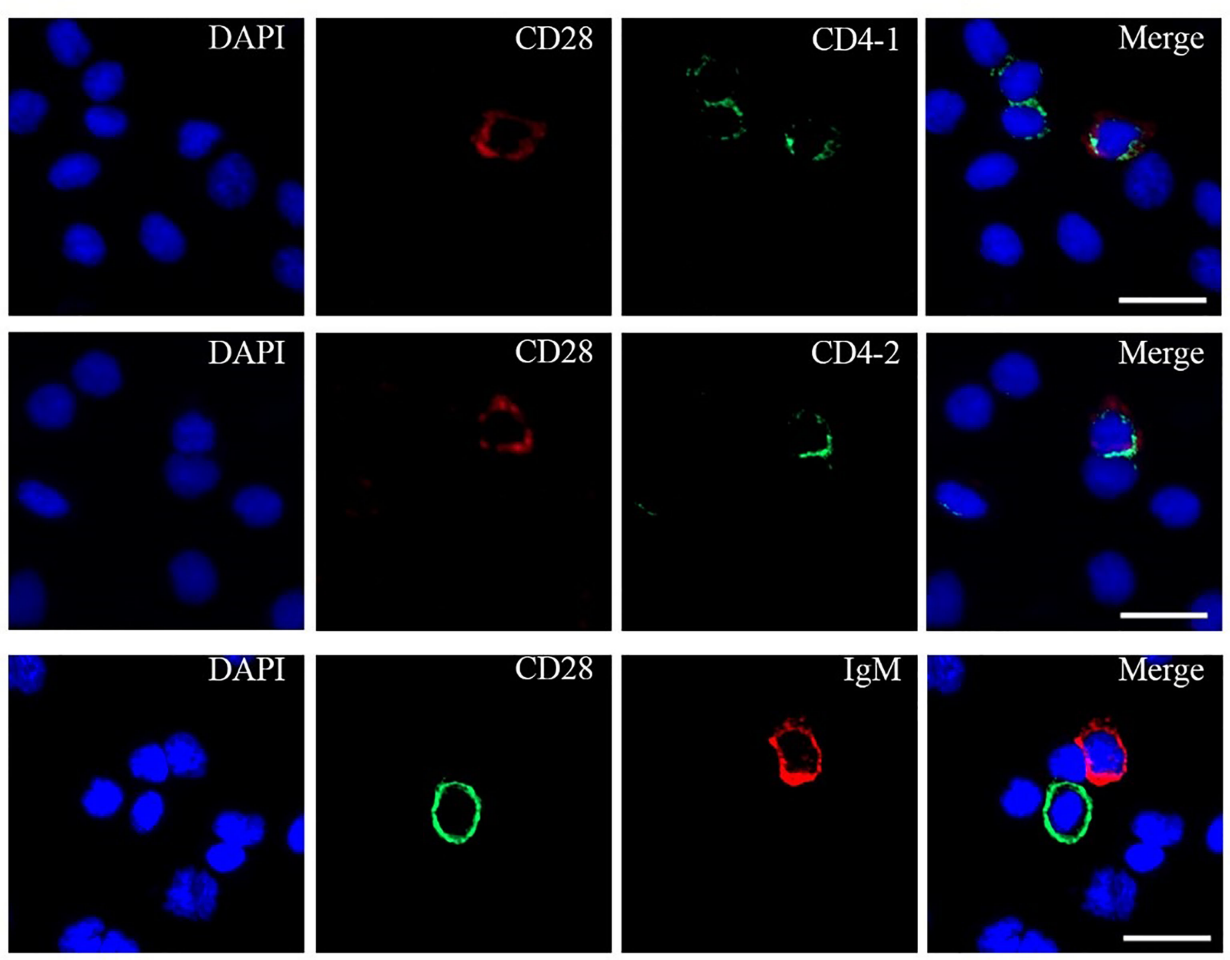
Co-localizations of CD28 with CD4-1, CD4-2, or IgM in PBLs. The leukocytes were incubated with primary Abs rabbit-anti CD28 with mouse-anti IgM, mouse-anti CD4-1, or mouse-anti CD4-2, respectively. DAPI staining shows the location of the nucleus. The unimmunized serum was used as negative control (data not shown). Scale bar = 10 μm.

**Figure 5:**
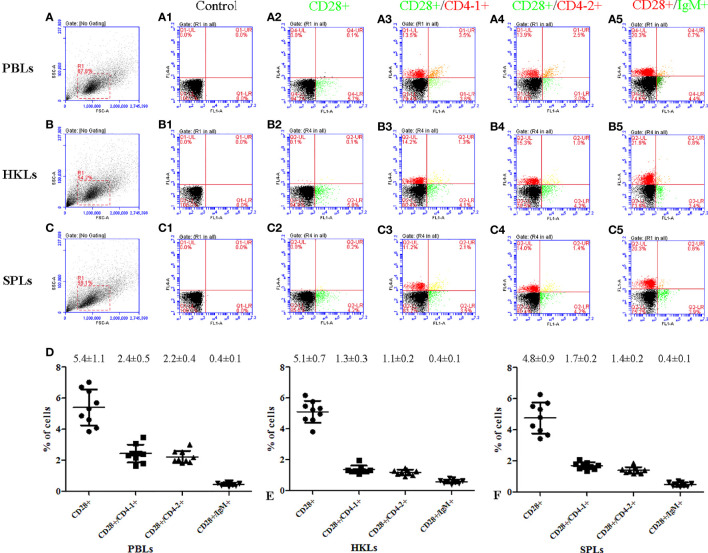
Flow cytometric analysis of CD28^+^, CD28^+^/CD4-1^+^, CD28^+^/CD4-2^+^, and CD28^+^/IgM^+^ leukocytes in peripheral blood, spleen, and head kidney. **(A–C)** FSC area (FSC-A)/SSC area (SSC-A) analyses and the gate represents leukocytes. **(A1–C1)** Negative controls using non-immunized mouse and rabbit serum as primary antibody; **(A2–C5)** rabbit anti-CD28 polyclonal antibody and mouse anti-CD4-1, CD4-2, and IgM monoclonal antibody were used as primary antibody; **(D)** percentages of CD28^+^, CD28^+^/CD4-1^+^, CD28^+^/CD4-2^+^, and CD28^+^/IgM^+^ leukocyte, data represent as mean ± SD, n = 9.

To further investigate the distribution of CD28 on leukocyte subsets, double-immunofluorescence staining was performed using the mouse anti-CD4-1, CD4-2, and IgM monoclonal antibodies prepared in the laboratory and rabbit anti-CD28 Abs as primary antibodies, respectively. The results showed that CD28 could be co-localized with CD4-1 or CD4-2 molecules on PBLs, respectively. However, no CD28-positive signal was detected on IgM^+^ cells, only the respective single positive signal (**Figure 4**). In addition, flow cytometric analysis also showed that the percentages of CD28^+^ cells were 5.4 ± 1.1% in PBLs, 5.1 ± 0.7% in HKLs, and 4.8 ± 0.9% in SPLs. Similarly, the percentages of CD28^+^/CD4-1^+^ cells, CD28^+^/CD4-2^+^ cells, and CD28^+^/IgM^+^ cells were 2.4 ± 0.5%, 2.2 ± 0.4%, and 0.4 ± 0.1% in PBLs; 1.3 ± 0.3%, 1.1 ± 0.2%, and 0.4 ± 0.1% in HKLs; 1.7 ± 0.2%, 1.4 ± 0.2%, and 0.4 ± 0.1% in SPLs (**Figure 5**). These observations suggested that CD28 is mainly expressed on T leukocytes and almost absent on B cells.

### Variation of CD28 ^+^ Cells in Flounder Post-Immunized With KLH and LPS

**Figure 6:**
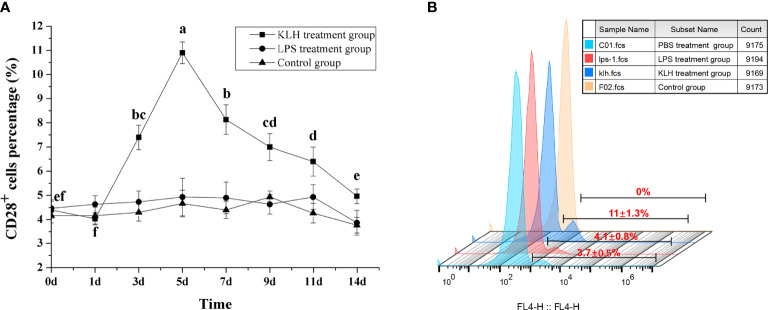
Percentages of CD28^+^ leukocytes in peripheral blood of flounder after injected with LPS or KLH determined by flow cytometry. **(A)** Variations of percentages of CD28^+^ leukocytes. Letters on the bar represent the statistically significant difference, *p* < 0.05, data are representative of nine individuals. **(B)** The fluorescent results of CD28^+^ leukocytes on the fifth day.

To compare the response pattern of CD28^+^ cells under different immunogenic stimuli, flounder were immunized with KLH and LPS, respectively. After stimulation with KLH, the percentage of CD28^+^ cells in PBLs increased on the third day (*p* < 0.05) and reached the peak on the fifth with 11.05 ± 1.2%, then the percentage was slowly decreased before decreasing to the level of the control group on the 14th day. In contrast, after stimulating with LPS, the percentage of CD28^+^ cells did not change significantly (*p* > 0.05), and there was no difference between the control groups (**Figure 6**). These different response patterns indicate that CD28 may play indispensable roles in T cell immune responses.

### The Proliferation of PBLs After Treatment Using PHA and Anti-CD28 *In Vitro*

**Figure 7:**
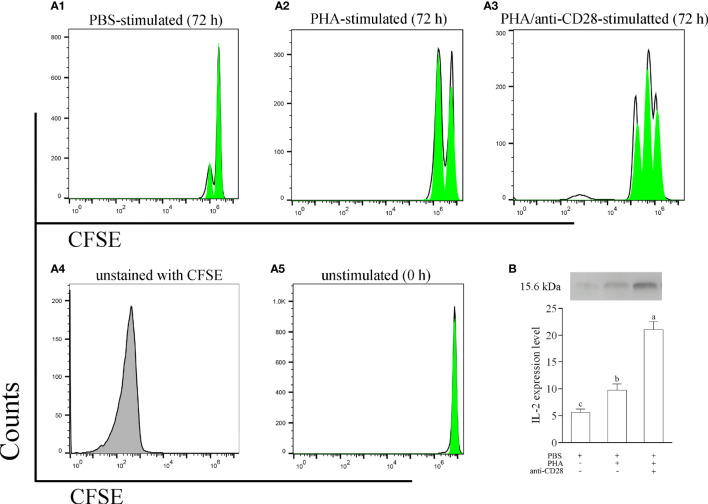
**(A)** The proliferation of leukocytes was monitored using CFSE labeling. CFSE-labeled PBLs (5 × 10^6^ cell/well) were treated with L-15 medium in the presence of PBS, PHA, or PHA+anti-CD28 were analyzed at 72 h of culture; data are representative of nine individuals. **(B)** The level of IL-2 in the culture medium at 72 h was measured by Western blot. The PBLs were stimulated with PBS, PHA, and PHA/anti-CD28, respectively. The experiment was repeated independently three times. Letters on the bar represent the statistically significant difference, *p* < 0.05.

To explore the role of the CD28 pathway in the function of leukocytes, a stimulation assay was performed with anti-CD28 Abs *in vitro*. As shown in **Figure S2** (in **Supplementary Material**), more colonies could be detected in cultured leukocytes with stimulation of PHA +anti-CD28. PHA also stimulated a clonal increase in leukocytes but less than the former, and then the changes in lymphocytes induced by PBS were not significant. Meanwhile, the divisions of CFSE-loaded PBLs were monitored by flow cytometry after 72 h of *in vitro* culture. Specifically, flow cytometry for leukocytes with PBS stimulation detected a single weaker peak of proliferation, the leukocytes with anti-CD28 stimulation were found to be able to generate three divisions, and the cells also generated two divisions with PHA stimulation (**Figure 7A**). Meanwhile, we also evaluated the effect of anti-CD28 Abs on IL-2 production.

The results revealed that the addition of anti-CD28 Abs significantly increased IL-2 secretion from PHA-stimulated lymphocytes after stimulation for 72 h (*p* < 0.05). Interestingly, a slight amount of IL-2 could be detected in the supernatant after PBS stimulation for 72 h (**Figure 7B**). These results suggest that anti-CD28 Abs may be involved in the activation of lymphocytes as a ligand for CD28. A non-related Abs, rabbit IgG, was used to replace anti-CD28 Abs, and the results of cell division showed no difference with the PBS-treated group (**Figure S3 in the Supplemental Material**).

### The Variations of CD28^+^ and CD4^+^ Cells After Treatment Using PHA and Anti-CD28 *In Vitro*

**Figure 8:**
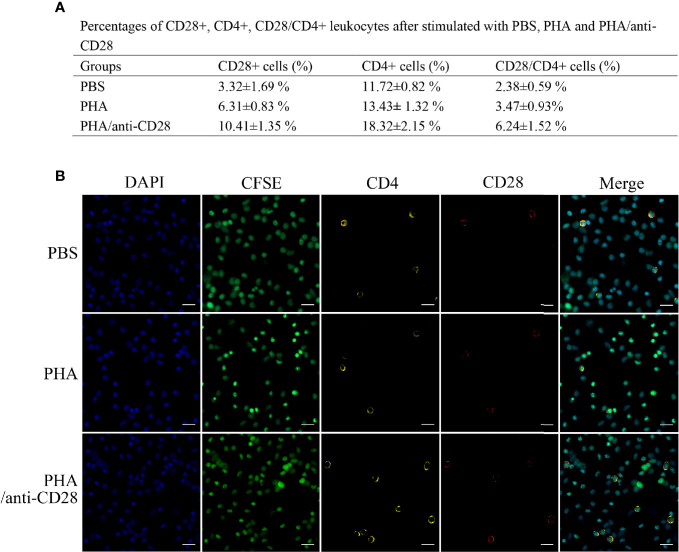
**(A)** The percentage of CD28+, CD4+, and CD28+/CD4+ PBLs after stimulation with PBS, PHA, and PHA/anti-CD28, respectively. **(B)** Co-localization of CD28+/CD4+ double-positive PBLs after stimulation with PBS, PHA, and PHA/anti-CD28, respectively. Indirect immunofluorescence detection of CFSE and CD28+/CD4+ cells of cultured PBLs after 72 h; data are representative of nine individuals; bar = 10 μm. The cell nuclei were counterstained in blue with DAPI. The unimmunized serum was used as the primary antibody for negative control (data not shown).

Flow cytometric analysis showed that the administration of anti-CD28 Abs promotes the percentage of CD4^+^ T lymphocytes (18.32 ± 2.15%, *p* < 0.05) compared with stimulation of PHA (13.43 **±** 1.32%) and PBS (11.72 ± 0.82%). The percentages of CD28^+^ cells in the PBS and PHA groups were 3.32 ± 1.69% and 6.31 ± 0.83%, whereas that in the group of PHA/anti-CD28 increased to 10.41 ± 1.35%. Meanwhile, the percentage of cells increased to 6.24 ± 1.52% (*p* < 0.05) in the PHA/anti-CD28 group (**Figure 8A**), and the proportion of CD28^+^ cells to CD4^+^ cells also increased from 20% (PBS group) to 34% (PHA/anti-CD28 group). These observations indicated that CD28^+^ and CD4^+^ cells increased as the cells proliferated with the stimulation of anti-CD28 Abs.

Fluorescence microscope observation showed that the green signals of CFSE were uniformly distributed in the cytoplasm. Double-immunofluorescence straining showed that CD4 could be co-located with CFSE and more positive signals could be detected in the PHA/anti-CD28 group. Moreover, the yellow fluorescent and red fluorescent corresponding to CD28 and CD4 were also detected on the CFSE-loaded cells. The distribution of CD28 and CD4 double-positive signals on CFSE-loaded cells with different optical density values may be related to the proliferation of the cells, and more CD28^+^/CD4^+^ positive signals showed in the PHA/anti-CD28 group which is consistent with that of FCM (**Figure 8B**).

### Expression of Genes After Treatment Using PHA and Anti-CD28 *In Vitro*

**Figure 9:**
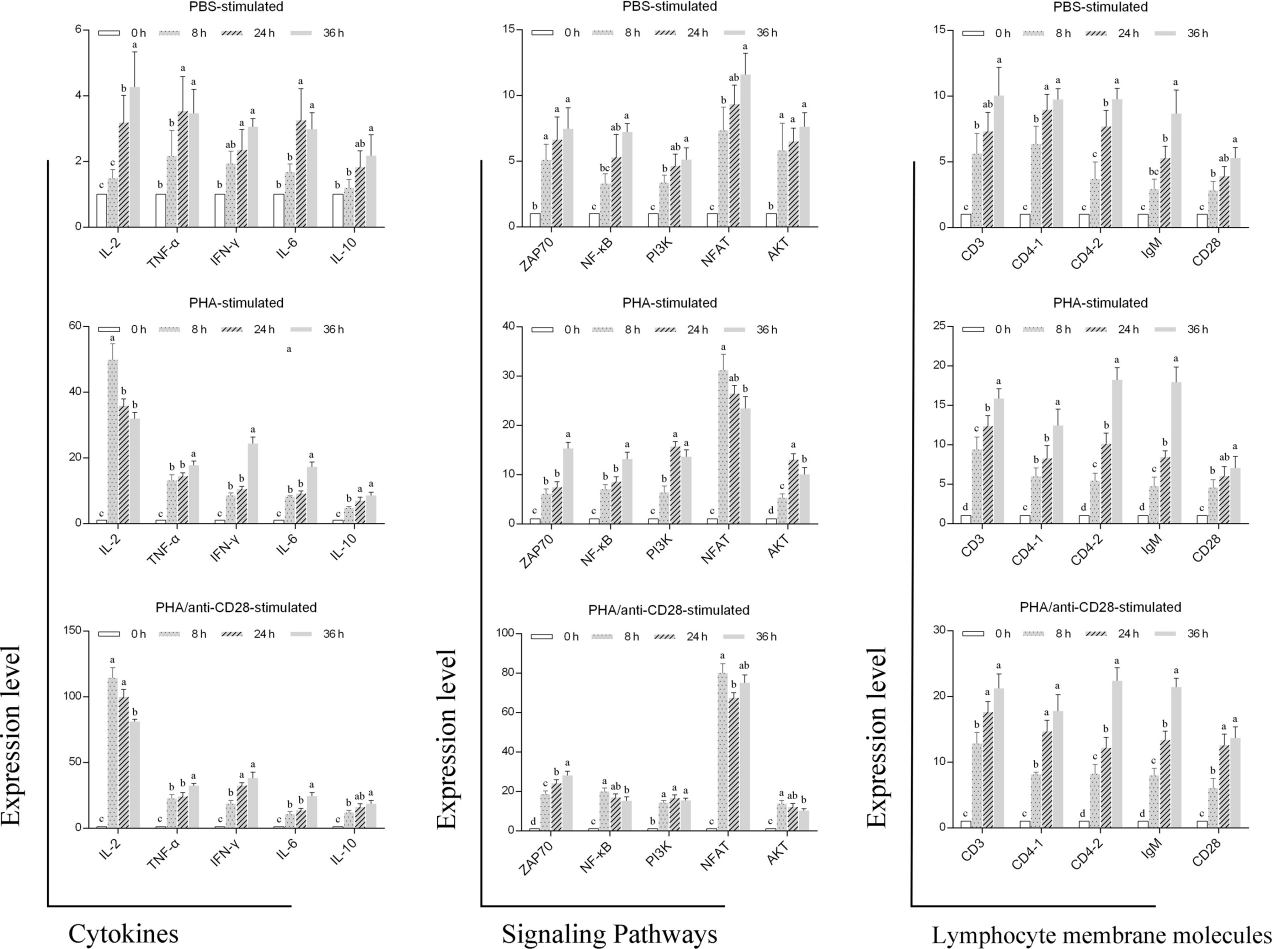
Gene expression. RT-PCR analysis of genes related to leukocyte membrane molecules, signaling pathways, and cytokines after 0, 8, 24, and 36 h of treatment of PBS, 5 μg/ml PHA or 5 μg/ml PHA +0.5 μg/ml anti-CD28 Abs. Data are expressed as SD. Data are from nine independent individuals; different letters represent the statistical significance (*p* < 0.05).

Compared with stimulation of PHA or PBS, the administration of anti-CD28 Abs with PHA strongly enhanced the expression of Th1-type cytokines including IL-2; IFN-γ and TNF-α were stronger than Th1-type cytokines of IL-6 and IL-10 in the early stages of culture. The highest *NFAT* expression was also monitored at 8 h post-stimulation, which is an immediate-early activation factor that plays a necessary early role in T cell activation. Furthermore, the expression of other genes was time-dependent, and the administration of anti-CD28 Abs significantly improved their level of expression. All detected genes also showed slight changes in the control group probably due to the normal growth process of the cells (**Figure 9**). These results may indicate that the CD28 signal pathway might participate in the Th1 immune response in fish.

## Discussion

CD28 engagement is indispensable for T cell activation. Ligation of CD28 to B7 ligands synergized with T cell receptor (TCR) initiates activation pathways including alterations in the threshold level of TCR ligation required for activation (7, 41), increases in cytokine secretion, particularly IL-2, prevention of anergy induction, and promotion of T cell survival. However, despite many CD28 homologies that have been identified, the distribution of CD28 on lymphocyte subsets and the relationship between T activation with the CD28 costimulatory pathway is still only partially understood in bony fish. In this study, the CD28 homolog was identified in flounder, and we have demonstrated the ability of the CD28 costimulatory pathway to enhance proliferative responses to PHA. These results will be useful in providing insights into the evolution of the adaptive immune responses of higher vertebrates.

CD28 in flounder comprised four exons and three introns, and the four exons encode the signal peptide, a superfamily Ig domain, the transmembrane region, and intracellular tail, respectively. Similarly, previous studies reported that intron/exon organization is consistent in bony fish and mammals such as half-smooth tongue sole, rainbow trout, medaka, zebrafish, European sea bass, and humans (29, 31, 42). Although the sequence identity of amino acid sequence comparisons between bony fish and mammals was less than 50%, the motifs responsible for signal reception and signal transduction are conserved in the CD28 sequence of flounder. The potential B7-binding motif TFPPPF in flounder is found in the Ig V-like domain, and different flanking residues of the PPP motif has been found in CD28 counterparts among vertebrates in the forms of KFPPPF (*O. latipes*), TYPPPY (*O. fasciatus*), MYPPPI (*O. mykiss*), SYPPPF (*D. rerio*), MFPPPL (*T. rubripes*), and MYPPPY (*H. sapiens and R. norvegicus*), which indicated that CD28 in flounder may share a similar signal recognition mechanism with higher vertebrates. Bernard *et al.* describe in their study that the YMDI motif corresponding to the YMNM motif in humans is presented in the cytoplasmic tail and confirmed that stimulation of the chimeric hCD28-rbtCD28 receptor promoted IL-2 production and induced the phosphorylation of the MAPK/ERK pathway (32). The cytoplasmic tail of the CD28 sequence in flounder also contains a ^199^YMNT^202^ motif, and the tyrosine phosphorylation site was found in the “YMNT” motif, which may suggest that this motif may be a potential PI3K-binding site for signal transduction. Those structural features support that the cloned CD28 in flounder is homologous to their counterparts.

Tissue distribution analysis shows that *fCD28* is constitutively highly expressed in gills, PBLs, head kidney, and spleen. Jeswin *et al.* point that RbCD28 is constitutively expressed in most tissues with a relatively higher expression in spleens, gills, trunk kidneys, and skin (34). Similarly, CD28 is constitutively expressed in all detected tissues of European sea bass with high expression in the thymus, spleen, and head kidney. However, the expression level of *CD28* in the gill is lower than those of the spleen and kidney in *C. semilaevis* (29). The spleen and head kidney usually serve as lymphoid organs in fish, and our previous studies have proven that many immune cells such as IgM+ B cells and CD4/CD8+ T cells can be found in these organs (39, 43–45). Although CD28 is constitutively expressed, their high expression in immune tissues implies their critical role in the immune response.

CD28 is mainly expressed on T lineage cells, including thymocytes, but may also be found on plasmablasts. Moreover, CD28 is expressed at higher levels on activated T cells than on resting cells (46, 47). Current studies have demonstrated that CD28 is expressed on the cell surface in *O. niloticus* and tongue sole (29, 30). In this study, the CD28 positive signal was also detected on the cell membrane of PBLs, and double immunofluorescence staining and FCM results showed that CD28+ cells were co-localized with CD4+ T lymphocytes but not on IgM+ B lymphocyte cells. It was reported that CD28 is expressed on 50% of CD8+ T cells and more than 80% CD4+ T cells in humans, while the percentage of CD28+ in CD4+ T subpopulations is around 20%. This may be related to the diversity of CD28 and lymphocyte subpopulation changes in fish, which needs to be further explored. We firstly reported that CD28 is mainly expressed on T lymphocytes in teleost which may indicate that CD28 may also act as a costimulatory receptor involved in adaptive immune.

To investigate whether flounder CD28 participates in antigen-induced immune response, the expression patterns were analyzed after being stimulated with KLH that require T cells to cooperate with B cells to synthesize specific antibodies (36, 48), as well as LPS which from gram-negative microorganisms is usually used to induce IgM synthesis by B cells without cooperation by T cells (49). In this study, the expression of *fCD28* was significantly upregulated by TD Ag (KLH) and the percentages of CD28+ cells in PBLs were significantly influenced by KLH stimulation, while no significant differences were observed between LPS stimulation. KLH is extensively used as the immunogen to assess the response of T cells in animal experiments (50, 51). In addition, González-Fernández *et al.* have reported that CD28 in the European sea bass was upregulated in head-kidney leucocytes after being stimulated with concanavalin A and PHA (T mitogens), but not with the B cell mitogen LPS (31). As our previous studies showed, the significant variations of T cells can be detected in the early post-stimulation period after being stimulated with KLH (35, 52, 53). The TD Ag upregulated expression of CD28 in fish suggested that CD28 may be closely associated with Th-mediated immune response.

T cell activation is a crucial event in the adaptive immunity. Engagement of the CD28 molecule with its ligand B7 is an essential regulator of CD4 T cell responses and ultimately leads to dramatically activated proliferation, cytokine production, and effector function in the T subset (54, 55). Recently, the methods of T cell activation based on the CD28 signaling pathway in mammals have been widely used (22, 23, 56), while the research about T cell activation in fish remains limited. In the present study, PHA and anti-CD28 Abs were used to simulate the stimulation of the TCR and CD28 signaling pathway in flounder. In our *in vitro* model, the supplementation with anti-CD28 Abs significantly enhanced PHA-induced T cell proliferation, and more cell colonies and cell division can be detected after being stimulated with PHA/anti-CD28 Abs. Although PHA is a selective T cell mitogen commonly used to assess T-cell proliferation, it may have a different effect than anti-CD3 on the TCR, thus producing a weaker proliferative effect. However, the effect of KLH/LPS in APC-initiated CD4+ T cell proliferation was significant in the zebrafish model, which may be associated with the different pathways triggering T cell activation (57). A similar report has also been confirmed in *C. semilaevis*, ligation of CsCD28 with and anti-rCsCD28 serum in cultured HKLs could induce significant levels of cellular proliferation (29), and IgG from unimmured rabbit was used to replace anti-CD28 Abs, and the results of cell division showed no difference with the PBS-treated group. Although the functional studies of CD28 in fish are limited, it can be assumed that CD28 plays an important role in the activation of fish lymphocytes like in mammals. However, ligation of CD28 with polyclonal antibodies induced lymphocyte proliferation in fish, and the proliferation ability of PHA/anti-CD28 is weaker compared with the effect of anti-CD3/anti-CD28 monoclonal antibodies on T cell proliferation in mammals (22). It is speculated that this may be related to the specificity of the antibody, and the specific mechanism of action deserves further study.

The secretion of cytokines is also a hallmark event of T cell activation, and T-cell activation signals induce the expression of several lymphokine genes, including IL-2, IFN-γ, and granulocyte-macrophage colony-stimulating factor (GM-CSF), IL-3 (58). In this study, the peak expression of IL-2 was reached at 8 h after stimulation, and the expression level in the PHA/anti-CD28 group is higher than in the control group. In addition, the results showed that the expression of Th1-type cytokines was earlier than and higher than Th2-type cytokines after anti-CD28 stimulation. Ai *et al.* found that after a 10-h culture, PHA increases the secretion of IL-2, IL-13, and RANTES in whole blood, but not IFN-γ, IL-10, IL-4, IL-6, and TNF-α (59). It is speculated that this difference may be related to the activation of the co-stimulatory signaling pathway. Meanwhile, the nuclear factor of activated T cells (NFAT) is an immediate-early activation factor that plays a necessary early role in T cell activation and commitment processes through its control of IL-2 gene activation (60). The highest upregulated expression of NFAT was also detected at 8 h after being stimulated with anti-CD28, and its early high expression may be involved in the induced expression of IL-2. In addition, the expression of fCD28 increased with time, while in the PHA/anti-CD28-stimulated group, the expression of fCD28 was not significantly different at 24 h and 36 h (*p > 0.05*), and significantly different (*p < 0.05*) compared with that at 8 h. This is similar to the results of KLH stimulation *in vivo*. It is speculated that the expression difference may be related to the first signal from PHA delivery causing T cell activation and thus contributing to CD28 expression. These results demonstrated that anti-CD28 Abs in flounder caused the early expression of NFAT and IL-2, which in turn affected the degree of lymphocyte activation and proliferation.

In summary, data presented here showed that the cloned CD28 in flounder is conserved to its homologs in fish and mammals, firstly clarified its distribution characteristics in fish lymphocyte subsets, and proved that the CD28 signaling pathway is a synergist to the PHA-induced lymphocyte activation. Those data lay a foundation for further exploring T lymphocyte activation in fish, and more studies are needed to reveal the necessity of co-stimulatory signaling pathways in fish T cell activation.

## Data Availability Statement

The datasets presented in this study can be found in online repositories. The names of the repository/repositories and accession number(s) can be found in the article/**Supplementary Material**.

## Ethics Statement

The animal study was reviewed and approved by the Guide for the Use of Experimental Animals of the Ocean University of China in agreement with the International Guiding Principles for Biomedical Research Involving Animals (EU84 2010/63).

## Author Contributions

Designed the experiments: WL, JX, and WZ. Performed the experiments: WL. Analyzed the data: WL, HC, and JX. Provided reagents/materials/analysis tools: XT and XS. Wrote the manuscript: WL, JX, and WZ. All authors participated in the revision of the manuscript and confirmed the integrity of this work. All authors contributed to the article and approved the submitted version.

## Funding

This work was supported by the National Key Research and Development Program of China (2018YFD0900503), the National Natural Science Foundation of China (32173005, 31730101, 31672684) and the Shandong Provincial Natural Science Foundation (ZR2020KC025).

## Conflict of Interest

The authors declare that the research was conducted in the absence of any commercial or financial relationships that could be construed as a potential conflict of interest.

## Publisher’s Note

All claims expressed in this article are solely those of the authors and do not necessarily represent those of their affiliated organizations, or those of the publisher, the editors and the reviewers. Any product that may be evaluated in this article, or claim that may be made by its manufacturer, is not guaranteed or endorsed by the publisher.

## References

[1] Marshall JS Warrington R Watson W Kim HL. An Introduction to Immunology and Immunopathology. Allergy Asthma Clin Immunol (2018) 14:1–10. doi: 10.1186/s13223-018-0278-1 30263032 PMC6156898

[2] Lees JR. CD8+ T Cells: The Past and Future of Immune Regulation. Cell Immunol (2020) 357:104212–9. doi: 10.1016/j.cellimm.2020.104212 32979764

[3] Whitmire JK Flavell RA Grewal IS Larsen CP Pearson TC Ahmed R. CD40-CD40 Ligand Costimulation is Required for Generating Antiviral CD4 T Cell Responses But is Dispensable for CD8 T Cell Responses. J Immunol (1999) 163:3194–201. 10477587

[4] Attanasio J Wherry EJ. Costimulatory and Coinhibitory Receptor Pathways in Infectious Disease. Immunity (2016) 44:1052–68. doi: 10.1016/j.immuni.2016.04.022 PMC4873956 27192569

[5] Schwartz RH. T Cell Anergy. Annu Rev Immunol (2003) 21:305–34. doi: 10.1146/annurev.immunol.21.120601.141110 12471050

[6] Wells AD. New Insights Into the Molecular Basis of T Cell Anergy: Anergy Factors, Avoidance Sensors, and Epigenetic Imprinting. J Immunol (2009) 182:7331–41. doi: 10.4049/jimmunol.0803917 19494254

[7] Riha P Rudd CE. CD28 Co-Signaling in the Adaptive Immune Response. Self/Nonself Immune Recognit Signal (2010) 1:231–40. doi: 10.4161/self.1.3.12968 PMC3047785 21487479

[8] Boomer JS Green JM. An Enigmatic Tail of CD28 Signaling. Cold Spring Harb Perspect Biol (2010) 2:1–20. doi: 10.1101/cshperspect.a002436 PMC2908766 20534709

[9] Esensten JH Helou YA Chopra G Weiss A Bluestone JA. CD28 Costimulation: From Mechanism to Therapy. Immunity (2016) 44:973–88. doi: 10.1016/j.immuni.2016.04.020 PMC4932896 27192564

[10] Lenschow DJ Walunas TL Jeffrey A. Cd28/B7 System of T Cell. Annu Rev Immunol (1996) 14:233–58. doi: 10.1146/annurev.immunol.14.1.233 8717514

[11] Fuse S Tsai CY Rommereim LM Zhang W Usherwood EJ. Differential Requirements for CD80/86-CD28 Costimulation in Primary and Memory CD4 T Cell Responses to Vaccinia Virus. Cell Immunol (2011) 266:130–4. doi: 10.1016/j.cellimm.2010.09.008 PMC3053005 21040905

[12] Hosseini A Gharibi T Marofi F Babaloo Z Baradaran B. CTLA-4: From Mechanism to Autoimmune Therapy. Int Immunopharmacol (2020) 80:106221–36. doi: 10.1016/j.intimp.2020.106221 32007707

[13] Iiyama M Numoto N Ogawa S Kuroda M Morii H Abe R. Molecular Interactions of the CTLA-4 Cytoplasmic Region With the Phosphoinositide 3-Kinase SH2 Domains. Mol Immunol (2021) 131:51–9. doi: 10.1016/j.molimm.2020.12.002 33386150

[14] Boise LH Minn AJ Noel PJ June CH Accavitti MA Lindsten T. CD28 Costimulation can Promote T Cell Survival by Enhancing the Expression of Bcl-XL. J Immunol (2010) 185:3788–99. doi: 10.1016/1074-7613(95)90161-2 20858890

[15] Zeng H Chi H. mTOR Signaling in the Differentiation and Function of Regulatory and Effector T Cells. Curr Opin Immunol (2017) 46:103–11. doi: 10.1016/j.coi.2017.04.005 PMC5554750 28535458

[16] Dodson LF Boomer JS Deppong CM Shah DD Sim J Bricker TL. Targeted Knock-In Mice Expressing Mutations of CD28 Reveal an Essential Pathway for Costimulation. Mol Cell Biol (2009) 29:3710–21. doi: 10.1128/mcb.01869-08 PMC2698768 19398586

[17] Téllez CN Siachoque JJ Siachoque SJ Siachoque JMA. Siachoque MH. T-Cell Activation, Alterations in Systemic Lupus Erythematosus: A Narrative Review. Rev Colomb Reumatol (English Ed (2018) 25:38–54. doi: 10.1016/j.rcreue.2018.09.001

[18] Chandler NJ Call MJ Call ME. T Cell Activation Machinery: Form and Function in Natural and Engineered Immune Receptors. Int J Mol Sci (2020) 21:1–25. doi: 10.3390/ijms21197424 PMC7582382 33050044

[19] Vallejo AN Brandes JC Weyand CM Goronzy JJ. Modulation of CD28 Expression: Distinct Regulatory Pathways During Activation and Replicative Senescence. J Immunol (1999) 162:6572–9. 10352273

[20] Beyersdorf N Hanke T Kerkau T Hünig T. Superagonistic Anti-CD28 Antibodies: Potent Activators of Regulatory T Cells for the Therapy of Autoimmune Diseases. Ann Rheum Dis (2005) 64:91–5. doi: 10.1136/ard.2005.042564 PMC1766908 16239397

[21] Lawlor N Nehar-Belaid D Grassmann JDS Stoeckius M Smibert P Stitzel ML. Single Cell Analysis of Blood Mononuclear Cells Stimulated Through Either LPS or Anti-CD3 and Anti-Cd28. Front Immunol (2021) 12:636720. doi: 10.3389/fimmu.2021.636720 33815388 PMC8010670

[22] Lustig A Manor T Liu Y-T Shi G Weng N. Anti-CD3 and Anti-CD28 Antibodies Conjugated With Lipid Microbubbles (MB-TAC) Provide a Superior Expansion of Human Naive CD4+ and CD8+ T Cells in Long Term Culture. J Immunol (2020) 204:475–84. doi: 10.4049/immunohorizons.2000056 PMC10433792 32769179

[23] Annette T Yiu Lam K. T Cell Stimulation and Expansion Using Anti-CD3/CD28 Beads. J Immunol Methods (2003) 275:251–5. doi: 10.1016/S0022-1759(03)00010-3 12667688

[24] Green L. Development of an Anti-CD2/CD3/CD28 Bead-Based T-Cell Proliferation Assay. Biosci Horizons Int J Student Res (2014) 7:2014. doi: 10.1093/BIOHORIZONS/HZU012

[25] Duarte RF Chen FE Lowdell MW Potter MN Lamana ML Prentice HG. Functional Impairment of Human T-Lymphocytes Following PHA-Induced Expansion and Retroviral Transduction: Implications for Gene Therapy. Gene Ther (2002) 9:1359–68. doi: 10.1038/sj.gt.3301807 12365001

[26] Yoshiaki A Chika Y Takuya M Takuya I Toshimitsu U Tsukasa M. Concanavalin A-Mediated T Cell Proliferation is Regulated by Herpes Virus Entry Mediator Costimulatory Molecule. In Vitro Cell Dev Biol Anim (2014) 50:313–20. doi: 10.1007/S11626-013-9705-2 24163161

[27] Sunyer JO. Fishing for Mammalian Paradigms in the Teleost Immune System. Nat Immunol (2013) 14:320. doi: 10.1038/NI.2549 23507645 PMC4203445

[28] Dickerson HW Findly RC. Vertebrate Adaptive Immunity-Comparative Insights From a Teleost Model. Front Immunol (2017) 8:1379. doi: 10.3389/fimmu.2017.01379 29123524 PMC5662878

[29] Hu YH Sun BG Deng T Sun L. Molecular Characterization of Cynoglossus Semilaevis CD28. Fish Shellfish Immunol (2012) 32:934–8. doi: 10.1016/j.fsi.2012.02.021 22530241

[30] Huang Y Wang Z Zheng Q Tang J Cai J Lu Y. Conservation of Structural and Interactional Features of CD28 and CD80/86 Molecules From Nile Tilapia (Oreochromis Niloticus). Fish Shellfish Immunol (2018) 72:95–103. doi: 10.1016/j.fsi.2017.10.008 29074133

[31] González-Fernández C Esteban MA Cuesta A. Molecular Characterization of the T Cell Costimulatory Receptors CD28 and CTLA4 in the European Sea Bass. Fish Shellfish Immunol (2021) 109:106–15. doi: 10.1016/j.fsi.2020.12.006 33348036

[32] Bernard D Riteau B Hansen JD Phillips RB Michel F Boudinot P. Costimulatory Receptors in a Teleost Fish: Typical CD28, Elusive Ctla4. J Immunol (2006) 176:4191–200. doi: 10.4049/jimmunol.176.7.4191 16547256

[33] Fang D-A Zhao C-S Jiang S-L Zhou Y-F Xu D-P. Toxic Function of CD28 Involving in the TLR/MyD88 Signal Pathway in the River Pufferfish (Takifugu Obscurus) After Exposed to Tributyltin Chloride (TBT-Cl). Gene (2019) 688:84–92. doi: 10.1016/J.GENE.2018.11.087 30529248

[34] Jeswin J Jeong SM Jeong JM Bae JS Kim MC Kim DH. Molecular Characterization of a T Cell Co-Stimulatory Receptor, CD28 of Rock Bream (Oplegnathus Fasciatus): Transcriptional Expression During Bacterial and Viral Stimulation. Fish Shellfish Immunol (2017) 66:354–9. doi: 10.1016/j.fsi.2017.05.013 28478261

[35] Xing J Luo K Xiao Y Tang X Zhan W. Influence of CD4-1+, CD4-2+ and CD8+ T Lymphocytes Subpopulations on the Immune Response of B Lymphocytes in Flounder (Paralichthys Olivaceus) Immunized With Thymus-Dependent or Thymus-Independent Antigen. Fish Shellfish Immunol (2019) 84:979–86. doi: 10.1016/j.fsi.2018.11.004 30395993

[36] Peachee VL Smith MJ Beck MJ Stump DG White KL. Characterization of the T-Dependent Antibody Response (TDAR) to Keyhole Limpet Hemocyanin (KLH) in the Göttingen Minipig. J Immunotoxicol (2014) 11:376–82. doi: 10.3109/1547691X.2013.853716 24219298

[37] Kasai H Yoshimizu M. Establishment of Two Japanese Flounder Embryo Cell Lines. Bull Fish Sci Hokkaido Univ (2001) 52:67–70.

[38] Xing J Tian HF Tang XQ Sheng XZ Zhan WB. Kinetics of T Lymphocyte Subsets and B Lymphocytes in Response to Immunostimulants in Flounder (Paralichthys Olivaceus): Implications for CD4+ T Lymphocyte Differentiation. Sci Rep (2020) 10:1–15. doi: 10.1038/s41598-020-69542-6 32796864 PMC7429840

[39] Li Q Zhan W Xing J Sheng X. Production, Characterisation and Applicability of Monoclonal Antibodies to Immunoglobulin of Japanese Flounder (Paralichthys Olivaceus). Fish Shellfish Immunol (2007) 23:982–90. doi: 10.1016/j.fsi.2007.03.008 17719797

[40] Zhou X Xing J Tang X Sheng X Chi H Zhan W. Interleukin-2 (IL-2) Interacts With IL-2 Receptor Beta (IL-2rβ): Its Potential to Enhance the Proliferation of CD4+ T Lymphocytes in Flounder (Paralichthys Olivaceus). Front Immunol (2020) 11:531785. doi: 10.3389/fimmu.2020.531785 33013923 PMC7509493

[41] Xia F Qian C-R Xun Z Hamon Y Sartre A-M Formisano A. TCR and CD28 Concomitant Stimulation Elicits a Distinctive Calcium Response in Naive T Cells. Front Immunol (2018) 0:2864. doi: 10.3389/FIMMU.2018.02864 PMC6288997 30564247

[42] Bernard D Hansen JD Du Pasquier L Lefranc MP Benmansour A Boudinot P. Costimulatory Receptors in Jawed Vertebrates: Conserved CD28, Odd CTLA4 and Multiple BTLAs. Dev Comp Immunol (2007) 31:255–71. doi: 10.1016/J.DCI.2006.06.003 16928399

[43] Bjørgen H Koppang EO. Anatomy of Teleost Fish Immune Structures and Organs. Immunogenetics (2021) 73:53–63. doi: 10.1007/s00251-020-01196-0 33426583 PMC7862538

[44] Xing J Ma J Tang X Sheng X Zhan W. Characterizations of CD4-1, CD4-2 and CD8β T Cell Subpopulations in Peripheral Blood Leucocytes, Spleen and Head Kidney of Japanese Flounder (Paralichthys Olivaceus). Mol Immunol (2017) 85:155–65. doi: 10.1016/j.molimm.2017.02.015 28260650

[45] Firdaus-Nawi M Zamri-Saad M. Major Components of Fish Immunity: A Review. Pertanika J Trop Agric Sci (2016) 39:393–420.

[46] Pellat-Deceunynck C Bataille R Robillard N Harousseau J Rapp M Juge- Morineau N. Expression of CD28 and CD40 in Human Myeloma Cells: A Comparative Study With Normal Plasma Cells. Blood (1994) 84:2597–603. doi: 10.1182/BLOOD.V84.8.2597.2597 7522634

[47] Mir MA. T-Cell Costimulation and Its Applications in Diseases. Dev Costimulatory Mol Immunother Dis (2015) 255–92. doi: 10.1016/B978-0-12-802585-7.00006-6

[48] AI L S Z P X. Characterization of Thymus-Dependent and Thymus-Independent Immunoglobulin Isotype Responses in Mice Using Enzyme-Linked Immunosorbent Assay. J Vis Exp (2018) 139:e57843–53. doi: 10.3791/57843 PMC6235121 30247482

[49] Obukhanych TV Nussenzweig MC. T-Independent Type II Immune Responses Generate Memory B Cells. J Exp Med (2006) 203:305. doi: 10.1084/JEM.20052036 16476769 PMC2118207

[50] Swaminathan A Lucas RM Dear K McMichael AJ. Keyhole Limpet Haemocyanin – A Model Antigen for Human Immunotoxicological Studies. Br J Clin Pharmacol (2014) 78:1135. doi: 10.1111/BCP.12422 24833186 PMC4243888

[51] Gong Y-F Xiang L-X Shao J-Z. CD154-CD40 Interactions Are Essential for Thymus-Dependent Antibody Production in Zebrafish: Insights Into the Origin of Costimulatory Pathway in Helper T Cell-Regulated Adaptive Immunity in Early Vertebrates. J Immunol (2009) 182:7749–62. doi: 10.4049/jimmunol.0804370 19494299

[52] Zhou X Xing J Tang X Sheng X Zhan W. Immunological Characteristics of Interleukin-2 Receptor Subunit Beta (IL-2rβ) in Flounder (Paralichtlys Olivaceus): Implication for IL-2R Function. Fish Shellfish Immunol (2019) 93:641–51. doi: 10.1016/j.fsi.2019.07.059 31344456

[53] Xing J Xiao Y Tang X Sheng X Zhan W. Inhibition of Cyclosporine A or Rapamycin on T Lymphocyte Counts and the Influence on the Immune Responses of B Lymphocytes in Flounder (Paralichthys Olivaceus). Fish Shellfish Immunol (2017) 66:78–85. doi: 10.1016/j.fsi.2017.05.017 28483552

[54] Glinos DA Soskic B Jostins L Sansom DM. Genomic Profiling of T Cell Activation Reveals Dependency of Memory T Cells on CD28 Costimulation. bioRvix (2018). doi: 10.1101/421099 PMC7785515 33223527

[55] Hwang JR Byeon Y Kim D Park SG. Recent Insights of T Cell Receptor-Mediated Signaling Pathways for T Cell Activation and Development. Exp Mol Med (2020) 52:750–61. doi: 10.1038/s12276-020-0435-8 PMC7272404 32439954

[56] KH R. Artificial Methods for T Cell Activation: Critical Tools in T Cell Biology and T Cell Immunotherapy. Adv Exp Med Biol (2018) 1064:207–19. doi: 10.1007/978-981-13-0445-3_13 30471035

[57] Shao T Shi W Zheng JY Xu XX Lin AF Xiang LX. Costimulatory Function of Cd58/Cd2 Interaction in Adaptive Humoral Immunity in a Zebrafish Model. Front Immunol (2018) 9:1204. doi: 10.3389/fimmu.2018.01204 29904386 PMC5990624

[58] Fraser JD Weiss A. Regulation of T-Cell Lymphokine Gene Transcription by the Accessory Molecule CD28. Mol Cell Biol (1992) 12:4357. doi: 10.1128/MCB.12.10.4357 1328852 PMC360359

[59] Ai W Li H Song N Li L Chen H. Optimal Method to Stimulate Cytokine Production and its Use in Immunotoxicity Assessment. Int J Environ Res Public Health (2013) 10:3834–42. doi: 10.3390/ijerph10093834 PMC3799516 23985769

[60] Hogan PG. Calcium–NFAT Transcriptional Signalling in T Cell Activation and T Cell Exhaustion. Cell Calcium (2017) 63:66. doi: 10.1016/J.CECA.2017.01.014 28153342 PMC5739523

