# Supplementary material for: The Expression of CD28 and Its Synergism on the Immune Response of Flounder (Paralichthys olivaceus) to Thymus-Dependent Antigen
Source: Front Immunol. 2021 Nov 11;12:765036. doi: 10.3389/fimmu.2021.765036 (PMC8631826; doi:10.3389/fimmu.2021.765036)
Supplement: Supplementary file 1 [file DataSheet_1.docx]

**The expression of CD28 and its synergism on the immune response of flounder (*Paralichthys olivaceus*) to thymus-dependent antigen**

Wenjing Liu^1^, Jing Xing^1,2*^, Xiaoqian Tang^1,2^, Xiuzhen Sheng^1^, Heng Chi^1^ and

Wenbin Zhan^1,2^

^1^ Laboratory of Pathology and Immunology of Aquatic Animals, KLMME, Ocean University of China, Qingdao, China,

^2^ Laboratory for Marine Fisheries Science and Food Production Processes, Qingdao National Laboratory for Marine Science and Technology, Qingdao, China

| **Supplemental Table S1**  Sequences of primers used for gene cloning. | | |
| --- | --- | --- |
| Primer name | Sequence (5'-3') | Application |
| *CD28-F1* | GTGCCGTGCCCAATCAAGACT | Gene cloning |
| *CD28-R1* | CTGCCCTCTGGGTTTGGTGTT |  |
| *CD28-1* | TAAAGGAGTGCCCAGTGTTGT | 3' RACE |
| *CD28-2* | GATTTGGATTCTGATGGTTGT |  |
| *CD28-1* | AGTCTTGATTGGGCACGGCAC | 5' RACE |
| *CD28-2* | CTTCACATCTGTAGGTTCCAT |  |
| *CD28-RFP-F* | CCCAAGCTTATGAGCGTTAGCTGGATGT (*HindIII*) | Eukaryotic expression |
| *CD28-RFP-R* | CGCGGATCCATGAAGTGTCGTGGTATTGGTTTC (*BamHI*) |  |
| *CD28-F2* | CCCAAGCTTTGCAGGTCATCATGTACCACTCATG (*BamHI*) | Prokaryotic expression |
| *CD28-R2* | CGCGGATCCTCAGAAGTGTCGTGGTATTGGTTTC (*HindIII*) |  |
| *CD28-F3* | CCCAATCAAGACTGAAGACGA | qPCR |
| *CD28-R3* | TAAAGGGAGGAGGGAATGTAA |  |
| *β-actin F* | GAGGGAAATCGTGCGTGACAT | qPCR |
| *β-actin R* | ATTGCCGATGGTGATGACCTG |  |
| F: forward; R: reverse; RACE, rapid amplification of cDNA ends; β-actin, accession No. HQ386788.1 | | |
|  | | |

|  |  |  |  |
| --- | --- | --- | --- |
| **Supplemental Table S2**  Sequences of primers used for RT-PCR | | | |
| Gene’s name | Primer sequences (5'-3') | Size (bp) | Genbank accession no. |
| ***CD3*** | **F:** ATCGCCATGACGCTCCTC | 161 | AB081751.1 |
|  | **R:** AAGCCTTTGGTTTGATCGTG |  |  |
| ***CD4-1*** | **F:** CAACTTGCCTGGCTCAATCACT | 213 | AB643634 |
|  | **R:** ACCGACTGGGTTCAAACTCACC |  |  |
| ***CD4-2*** | **F:** GGTCCGCACCTACAAGAATAGC | 165 | AB640684 |
|  | **R:** ATCCCACTGTCTTCGTTCCTCA |  |  |
| ***IgM*** | **F:** AAGTCCACAAATTACCCTCCAA | 219 | AB052744 |
|  | **R:** TTCTCGCTTTTATGTTCCTCAG |  |  |
| ***CD28*** | **F:** TTCCAACGTCTCATGCACTGG | 94 | MT019836.1 |
|  | **R:** TTTTTGCTGTTTGCGCTCCAC |  |  |
| ***IL-2*** | **F:** TAGAGGATGCCAGTATCGGTT | 246 | KY307833.1 |
|  | **R:** TACATTCTGCGGAGGTCGTTG |  |  |
| ***IL-10*** | **F:** CTACGAAGCGAACGATGA | 200 | XM_020086558.1 |
|  | **R:** CGAAGATTTGCTGTATGGA |  |  |
| ***IL-6*** | **F:** CAAAGGTTGGCTGAAGGC | 218 | DQ267937.1 |
|  | **R:** TGGAAAGTGCTGGGGTTG |  |  |
| ***TNF-α*** | **F:** TCCTGGCGTTTTCTTGGT | 135 | AB040448.1 |
|  | **R:** TGGCTCTGCTGCTGATTT |  |  |
| ***IFN-γ*** | **F:** TGGTCTGTCTGTCCCTGTG | 132 | AB435093.1 |
|  | **R:** GCTTCCCGTTGAATCTGT |  |  |
| ***AKT*** | **F:** TCGTAGCAAAAGATGAAGT | 164 | XM_020084943.1 |
|  | **R:** TGACAAGTGGAAGAAAAGC |  |  |
| ***PI3K*** | **F:** CCTTGGAAAGAAATGGAT | 153 | KY763984.1 |
|  | **R:** TCTGGAGGGGAAAACTGC |  |  |
| ***NF-κB*** | **F:** CAACAGAGCCCCAAACAC | 264 | HM771267.1 |
|  | **R:** CGGAGCTGCATCTTCACT |  |  |
| ***ZAP70*** | **F:** CTAGTGCGAGACAGAGATGA | 115 | XM_020098352.1 |
|  | **R:** CCTCTGGCATGGAGAATTTAC |  |  |
| ***NFAT*** | **F:** CCCGTGCATGTCAACTTCTACGT | 112 | XM_020091042.1 |
|  | **R:** CATCAGTGGGTTCTGTCTTTATTATCG |  |  |
| ***β-actin*** | **F:** GAGGGAAATCGTTCGTGACAT | 142 | AF135499.1 |
|  | **R:** ATTGCCGATGGTGATGACCTG |  |  |

**Supplemental Table S3**

| **Molecule** | **Species** | **Accession number** |
| --- | --- | --- |
| **CD28** | *H. sapiens* | CAD57003 |
|  | *M. mulatta* | ABH08508.1 |
|  | *C. familiaris* | AAF72533.1 |
|  | *R. norvegicus* | CAA39003.1 |
|  | *G.gallus* | NP_990642.1 |
|  | *S. alpinus* | XP_023828332.1 |
|  | *C. semilaevis* | AEM98131 |
|  | *O. niloticus* | AMQ36815.1 |
|  | *T. rubripes* | NP_001267008.1 |
|  | *O. fasciatus* | ARP51378.1 |
|  | *D. labrax* | AIK66542.1 |
|  | *P. olivaceus* | MT019836.1 |
| **CTLA-4** | *H. sapiens* | NP_005205.2 |
|  | *M. musculus* | NP_033973.2 |
|  | *C. idella* | ANS56464.1 |
|  | *O. mykiss* | NP_001118005.1 |


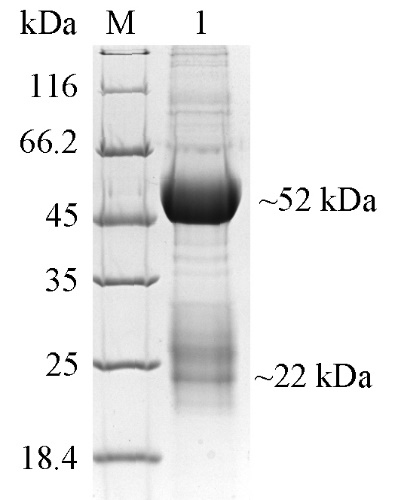


**Supplemental Figure S1**. SDS-PAGE analysis of purified anti-CD28 polyclonal antibody. Lane M, protein molecular weight marker; Lane 1, polyclonal antibody purified by HiTrap Protein G HP column.


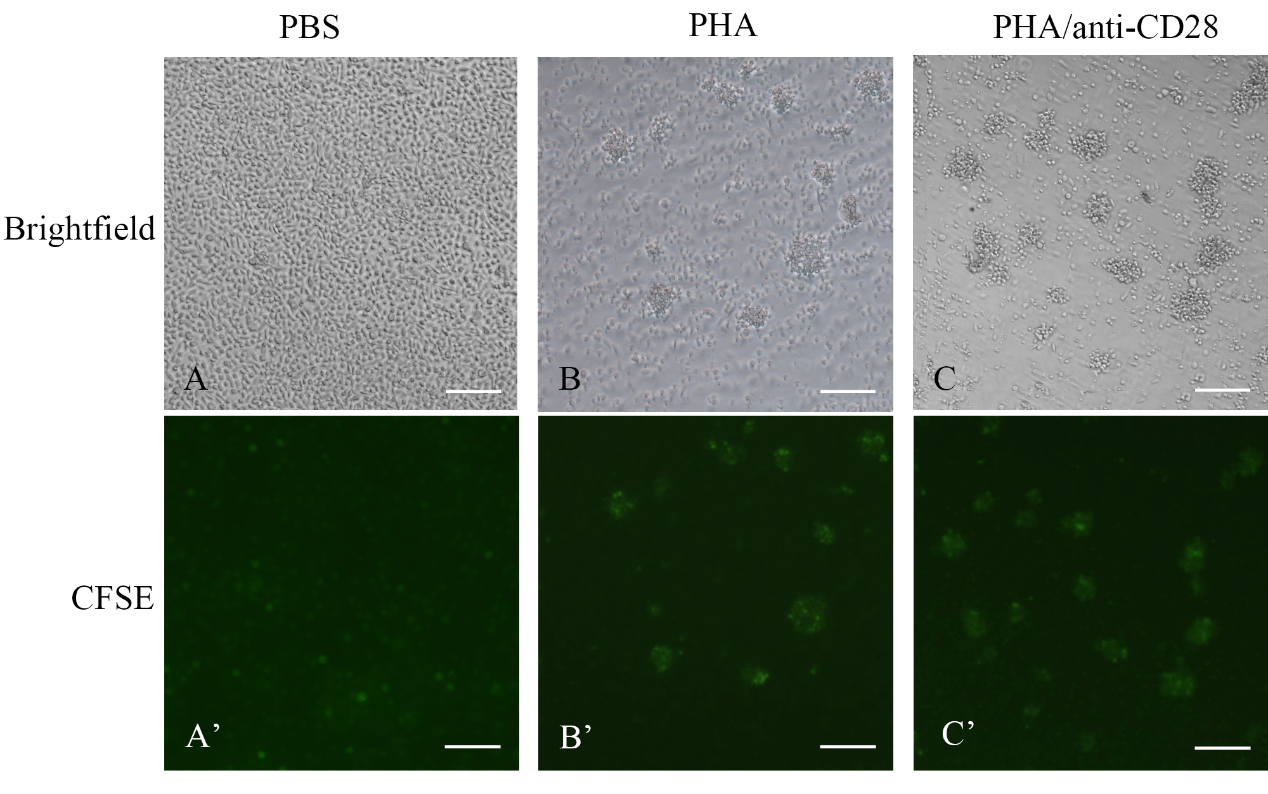


**Supplemental Figure S2**. Effect of stimulants on cultured leukocytes of flounder. Lymphocytes from peripheral blood were cultured for 72h with PBS (**A** and **A’**); with PHA (**B** and **B’**); with PHA+anti-CD28 (**C** and **C’**), respectively. Bar=50 μm. Images are representative of *n* =3.


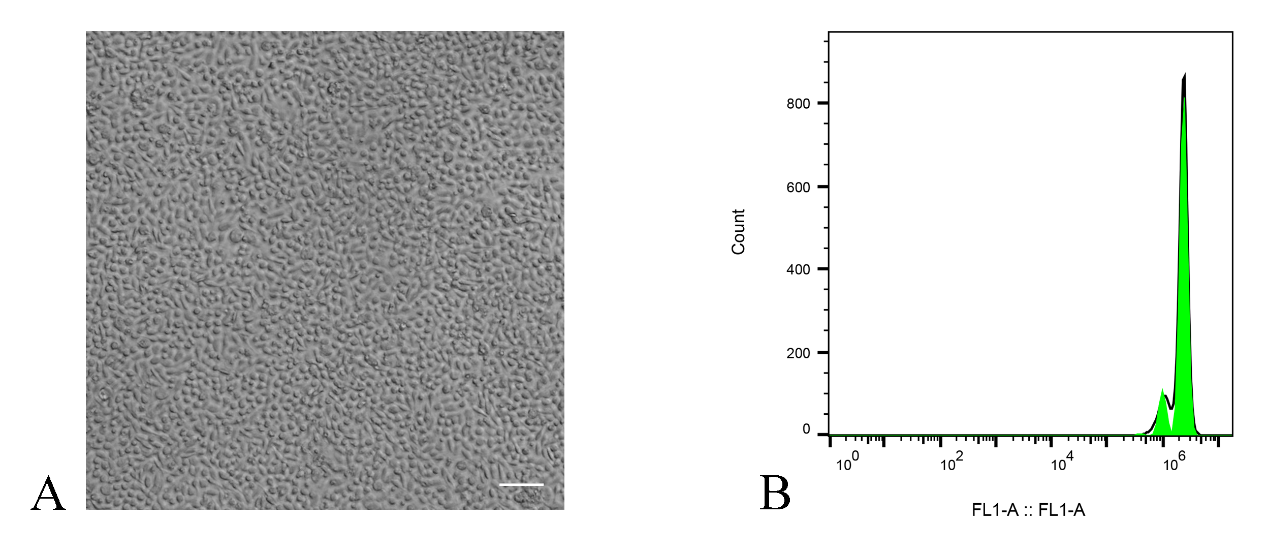


**Supplemental Figure S3.** Effect of un-immunized rabbit-IgG on cultured leukocytes of flounder. **A.** Lymphocytes from peripheral blood cultured for 72 h with of rabbit. Bar=50 μm. Images are representative of *n* =3. **B.** Leukocytes proliferation measured by CFSE. The leukocytes were cultured with un-immunized rabbit-IgG for 72 h, n=3.
